# Can Persistent Homology Features Capture More Intrinsic Information about Tumors from ^18^F-Fluorodeoxyglucose Positron Emission Tomography/Computed Tomography Images of Head and Neck Cancer Patients?

**DOI:** 10.3390/metabo12100972

**Published:** 2022-10-14

**Authors:** Quoc Cuong Le, Hidetaka Arimura, Kenta Ninomiya, Takumi Kodama, Tetsuhiro Moriyama

**Affiliations:** 1 Ho Chi Minh City Oncology Hospital, Ho Chi Minh City 700000, Vietnam; 2 Department of Health Sciences, Faculty of Medical Sciences, Kyushu University, Fukuoka City 812-8582, Japan; 3 Sanford Burnham Prebys Medical Discovery Institute, 10901 North Torrey Pines Road, La Jolla, San Diego, CA 92037, USA; 4 Department of Health Sciences, Graduate School of Medical Sciences, Kyushu University, Fukuoka City 812-8582, Japan; 5 Institute of Mathematics for Industry, Kyushu University, Fukuoka City 819-0395, Japan

**Keywords:** radiomics, persistent homology, head and neck cancer, prognostic prediction

## Abstract

This study hypothesized that persistent homology (PH) features could capture more intrinsic information about the metabolism and morphology of tumors from ^18^F-fluorodeoxyglucose positron emission tomography (PET)/computed tomography (CT) images of patients with head and neck (HN) cancer than other conventional features. PET/CT images and clinical variables of 207 patients were selected from the publicly available dataset of the Cancer Imaging Archive. PH images were generated from persistent diagrams obtained from PET/CT images. The PH features were derived from the PH PET/CT images. The signatures were constructed in a training cohort from features from CT, PET, PH-CT, and PH-PET images; clinical variables; and the combination of features and clinical variables. Signatures were evaluated using statistically significant differences (*p*-value, log-rank test) between survival curves for low- and high-risk groups and the C-index. In an independent test cohort, the signature consisting of PH-PET features and clinical variables exhibited the lowest log-rank *p*-value of 3.30 × 10^−5^ and C-index of 0.80, compared with log-rank *p*-values from 3.52 × 10^−2^ to 1.15 × 10^−4^ and C-indices from 0.34 to 0.79 for other signatures. This result suggests that PH features can capture the intrinsic information of tumors and predict prognosis in patients with HN cancer.

## 1. Introduction

Head-and-neck (HN) cancer is the sixth leading cancer worldwide [1]; more than 90% of the patients with HN cancer are diagnosed with HN squamous cell carcinoma (HNSCC), which is the focus of this study. The five-year survival rate of patients with HN cancer is only approximately 50% [2] because of distant metastasis and second primary cancers [3,4]. HN cancer patients still exhibit varied survival outcomes [5], implying that inappropriate treatments may have been administered to them. Therefore, prognostic prediction prior to treatment could facilitate personalized medicine administration and prolong survival.

Owing to the association of tumor genetic heterogeneity in HN cancer with patients’ prognoses, it was concluded that higher heterogeneity is related to worse outcomes [6,7,8]. This genetic heterogeneity can lead to imaging heterogeneity, which can be quantified by radiomics [9] on ^18^F-fluorodeoxyglucose (FDG) positron emission tomography (PET)/computed tomography (CT) images [10,11]. The feasibility of conventional PET/CT features for risk assessment in patients with HN cancer has been extensively investigated [12,13]. Due to relatively higher Kaplan–Meier *p*-values ≥ 10^−3^ in these studies, we believe that there is still room for developing new technologies for improving the prediction of prognoses in patients with HN cancer.

One of the novel theories for evaluating tumor heterogeneity is topology, which is a mathematical study of the geometrical properties of connectedness in objects [14]. The successful application of topology in lung cancer [15,16] motivated us to investigate its usefulness for treating HN cancer. Homology features of topological space X are encoded into homology group $H_{k}\left(X\right)$, whose rank is referred to as the *k*-dimensional Betti number. Betti numbers represent invariant properties of objects under continuous deformation, for example, the number of connected components and holes. Persistent homology (PH) is a fundamental tool in topological analysis to track the emergence (birth) and disappearance (death) of homology features (connected components and holes) in a nested sequence of data [17,18].

Since PH can exploit the hidden geometrical properties of data [17,18], we hypothesized that PH features could capture more intrinsic information on the metabolism and morphology of tumors that could be associated with prognoses on PET/CT images than conventional features. This study, thus, investigated the feasibility of using PH features for prognostic prediction of patients with HN cancer by using PET/CT images. To the best of our knowledge, this is the first study to examine the potential of PH features on PET/CT images for prognostic prediction of patients with HN cancer.

## 2. Materials and Methods

### 2.1. Clinical Cases

From a publicly available dataset [13], the Cancer Imaging Archive (https://www.cancerimagingarchive.net/, accessed on 11 July 2020), PET/CT images, and clinical variables of 207 patients with HNSCC were selected with careful anonymization. As this dataset was publicly available for research purposes, approval from the institutional review board was not required. The patients were treated with radiotherapy or concurrent chemoradiotherapy from 2006 to 2014 at four institutions in Canada [13]. Cancer staging was performed, according to the American Joint Committee on Cancer 7th edition. A total of 73 patients were set aside as a completely independent test cohort, whereas the remaining 134 patients formed a training cohort. This division follows the work of Vallières et al. [13]. The clinical variables of the patients included in this study are listed in Table 1.

Three FDG-PET/CT scanners (Discovery ST and Discovery STE, GE Healthcare, Fairfield, CT, USA; and GeminiGXL 16, Philips, Amsterdam, The Netherlands) were used for image acquisition. The size of CT images was 512 × 512 pixels with in-plane pixel sizes of 0.98–1.95 mm, slice thicknesses of 1.50–5.00 mm, X-ray tube voltages of 120–140 kV (median: 140 kV), and exposures of 29–469 mAs (median: 70 mAs). Contrast-enhanced CT images were available for 166 patients only. The images were reconstructed using B and C (Philips) and standard and soft kernels (GE Healthcare). For PET scans, FDG of 198–859 MBq (median 403 MBq) was injected intravenously [13]. The sizes of the PET images were 128 × 128 and 144 × 144 pixels with in-plane pixel sizes of 3.52–5.47 mm and slice thicknesses of 3.27–4.00 mm. PET images were acquired using multiple bed positions with a median of 300 s (range, 120–420 s) per bed position. Attenuated corrected images were reconstructed using an order subset expectation maximization iterative algorithm with a span (axial mash) of 5 (range: 3–5) and line-of-response row-action maximum likelihood algorithm. The filter cut-off, number of subsets, and iterations were not mentioned in the original paper [13]. Gross tumor volumes (GTVs) were delineated on different CT images dedicated to treatment planning by expert oncologists [13]. The contours were superimposed onto the PET/CT scanning coordinate system using deformable registration software (MIM Software Inc., Cleveland, OH, USA). The effective diameter of the GTVs ranged from 15.6 to 90.2 mm (mean: 42.9 mm). Anisotropic CT, PET images, and their corresponding GTVs were converted into isotropic images with isovoxel sizes of 0.98 mm for CT and 3.52 mm for PET, using cubic and shape-based interpolation [19].

Another publicly available dataset (RIDER), which consists of 28 sets of test–retest lung cancer CT images acquired approximately 15 min apart under the same imaging protocol [20], was used to examine the repeatability of the features extracted from the CT and PH images. Since radiomic features have been proven to be transferable from lung to HN cancer [10], this RIDER dataset could be appropriate for the examination of repeatability in our HN cancer study. The images were acquired using a 16- and 64-row scanner (LightSpeed 16 and VCT, GE Healthcare) with a tube voltage of 120 kV and tube current from 298 to 441 mA. The contours for the test and retest were produced in agreement with three radiologists with more than 10 years of experience with chest CT [20].

### 2.2. Overall Workflow

**Figure 1:**
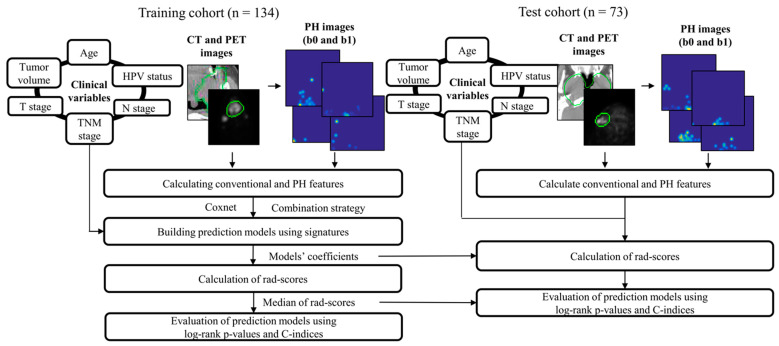
Overall workflow employed in this study. PH: persistence homology, HPV: human papilloma virus; CT: computed tomography; PET: positron emission tomography; rad-score: radiomic score; the green circle represents gross tumor volume.

Figure 1 illustrates the workflow of this study. PH images (b0 and b1) were first generated from CT and PET images. Conventional features were calculated from the CT, PET, and their wavelet-decomposed images. PH features were extracted from the b0 PH, b1 PH, and their wavelet-decomposed filtered images. Clinical variables (age, T stage, N stage, TNM stage, tumor volume, and human papilloma virus [HPV] status) were also investigated in terms of prognosis. Cox proportional hazard models (CPHMs) [21] were built for each signature using the Coxnet algorithm [22,23] and a combination strategy [24]. A radiomic score (rad-score) [12,22], that is, a linear combination of features in the signature weighted by their corresponding CPHM coefficients, was calculated for each patient. The patients were stratified into low- and high-risk groups for short-term survival based on the median of the rad-scores, and a log-rank *p*-value between the two survival curves and the C-index was calculated for model evaluation in the training cohort. The coefficients and medians of the rad-scores were fixed and applied to the test cohort for rad-score calculation and model evaluation, respectively.

Table 2 presents the details of all types of signatures constructed in this study. As clinical, conventional, PH, and combined signatures were constructed, there were 13 types of signatures in this study.

### 2.3. Persistent Homology Images

**Figure 2:**
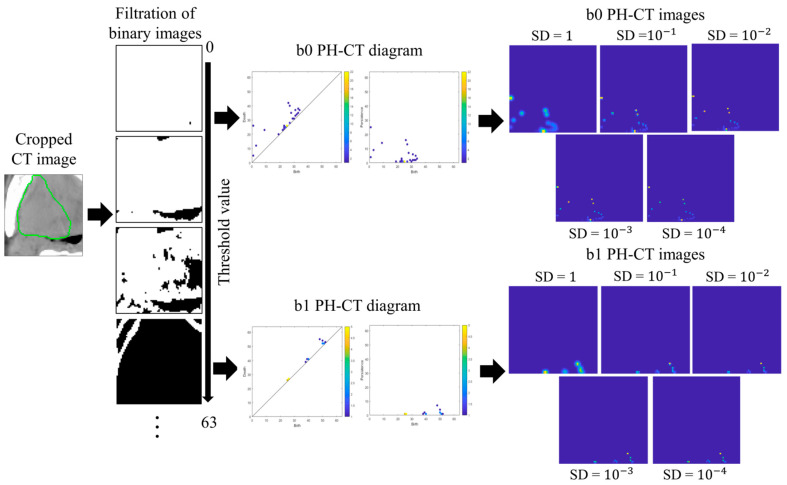
The process of generating PH-CT images in this study. CT: computed tomography; PH-CT: persistent homology-CT; the green circle represents gross tumor volume.

PH images were vectorized from PH diagrams [25,26,27]. Figure 2 illustrates the process of generating the b0 and b1 PH-CT images (the same for the b0 and b1 PH-PET images). CT images were cropped into a 10-voxel-large rectangular volume surrounding the GTVs; quantized into 6-, 7-, 8-, and 9-bit depths; and binarized by applying multiple thresholds to generate a filtration of the binary images [25]. A b0 PH diagram constituted of birth–death pairs of all connected components (black regions) that were generated and died within the filtration of binary images. A b1 PH diagram constituted birth–death pairs of holes (white regions containing no pixels at the edge). Black and white regions seem to be holes and connected components, respectively, in the original CT image in Figure 2, but we respectfully followed the definitions of the topological data analysis software HomCloud [26,27] employed for productions of PH diagrams in this study.

Let $B\left(b_{k},d_{k}\right)$ be a PH diagram in the birth–death coordinates $\left(b_{k},d_{k}\right),k\epsilon \left\{1,\;2,\ldots ,\;N\right\}$ where N denotes the number of pairs. $B\left(b_{k},d_{k}\right)$ was mapped into $B\left(b_{k},p_{k}=d_{k}-b_{k}\right)\;$ in the birth–persistence coordinates. Next, $B\left(b_{k},p_{k}\right)$ was transformed into PH images, $\rho_{B}\left(x,y\right)$. A PH image is a summation of multiple weighted Gaussian distributions centered at each coordinate on the diagram $B\left(b_{k},p_{k}\right)$. As CT and PET images of the same patient acquired at different times may vary, each point in the PH diagram may contain uncertainty. The use of Gaussian distributions can address this uncertainty and ensure stable transformation from PH diagrams to PH images [28]. Five standard deviation (SD) values of the Gaussian distribution (1, 10^−1^, 10^−2^, 10^−3^, and 10^−4^) were used. The Gaussian distribution can be expressed as:

(1) $$G_{B}{}_{\left(b_{k},p_{k}\right)}\left(x,y\right)=\frac{1}{2\pi \sigma^{2}}\exp\left[\frac{{\left(x-b_{k}\right)}^{2}+{\left(y-p_{k}\right)}^{2}}{2\sigma^{2}}\right]\times B\left(b_{k},p_{k}\right),$$

where σ denotes the SD, and x and y are the row and column of a pixel on the PH image, respectively.

A weighting function is essential for the stable transformation from PH diagrams to PH images [28]. In this study, a linear weighting function that can adjust the importance of pairs in different regions can be expressed as:

(2) $$w_{P}\left(p_{k}\right)=\{\begin{array}{c} 0,\;\;p_{k}\leq 0\\ \frac{p_{k}}{P},\;\;0<p_{k}<P\\ 1,\;\;p_{k}\geq P \end{array},$$

where P is the depth of the quantized CT or PET images (e.g., P = 255 if the images were 8-bit deep).

Therefore, the PH images [28] can be expressed as:

(3) $$\rho_{B}\left(x,y\right)=\overset{N}{\underset{k=1}{\sum }}w_{P}\left(p_{k}\right)G_{B}{}_{\left(b_{k},p_{k}\right)}\left(x,y\right)$$

**Figure 3:**
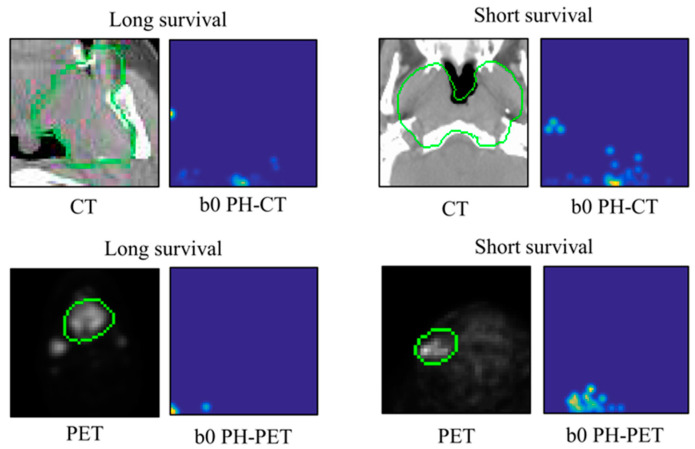
Illustrations of b0 PH-CT and PH-PET images for long- and short-survival patients. PH: persistent homology; CT: computed tomography; PET: positron emission tomography; the green circle represents gross tumor volume.

As the resolution may not affect classification task performance [28,29], a size of 64 × 64 pixels was fixed for all PH images. A Matlab-based package [28] was used for generating PH images in the Matlab 2018a environment. Figure 3 illustrates the examples of b0 PH-CT and b0 PH-PET images for long- and short-survival patients.

### 2.4. Calculating Conventional and Persistent Homology Features

Radiomic features (14 histograms, 45 textures, and 472 wavelets; Supplementary Table S1) were derived from the CT, PET, PH-CT, and PH-PET images. Histogram features were calculated using the original voxel values. Texture features were calculated using 6-, 7-, 8-, and 9-bit images, acquired based on the 0–63, 0–127, 0–255, and 0–511 look-up tables, respectively. Wavelet features were calculated from eight decomposed images of low (L) and/or high (H) frequency filters (LLL, HLL, LHL, HHL, LLH, HLH, LHH, and HHH) using a *coiflet* 1 mother wavelet. Radiomic features were calculated using a radiomic package [30] in the 2018a MATLAB environment (MathWorks, Natick, MA, USA).

The radiomic features were extracted from the RIDER dataset, and intraclass correlation coefficients (ICCs) were then calculated. An ICC < 0.5 indicated low repeatability; 0.5 ≤ ICC < 0.75, moderate repeatability; 0.75 ≤ ICC < 0.9, good repeatability; and ICC ≥ 0.9, excellent repeatability [31].

The conventional CT, PET, b0 PH-CT, b1 PH-CT, b0 PH-PET, and b1 PH-PET feature sets contained features extracted from CT, PET, b0 PH-CT, b1 PH-CT, b0 PH-PET, and b1 PH-PET images, respectively. Conventional CT and PET signatures were combined to form the conventional PET/CT feature set; b0 PH-CT and b0 PH-PET formed b0 PH-PET/CT; b1 PH-CT and b1 PH-PET formed b1 PH-PET/CT; and b0 and b1 PH (CT, PET, and PET/CT) formed b0 + b1 PH (CT, PET, and PET/CT).

### 2.5. Building Prediction Models Using Signatures

A Coxnet algorithm [22,23,32], whose α was optimized using a grid search, was used to select the signature candidates. The Coxnet algorithm was performed in R (R Core Team, Vienna, Austria) software version 3.6.3 using the function *glmnet* in the package *glmnet*. Clinical variables were converted into numeric values (Supplementary Table S2), and signature candidates were considered. HPV status, which was not fully available in our dataset (*n* = 83/207), was analyzed separately in univariate analysis. Tumor volume, an independent prognostic factor for HN cancer [33], was also analyzed separately.

The signatures were constructed using a combination strategy [24]. Thirteen signatures consisting of 1–13 features were constructed and 13 CPHMs were built. Care was taken that the number of features in the signature was not larger than one-tenth the size of the training cohort (*n* = 134) [34]. CPHMs were constructed in R using the *coxph* function in the *survival* package. Conventional and PH signatures were combined with clinical signatures. Thus, six combined signatures were constructed (Table 2) and six CPHMs were built.

### 2.6. Calculation of Rad-Scores and Evaluation of Prediction Models

The Kaplan–Meier procedure was used to evaluate all types of signatures (Table 2). Statistically significant differences (*p*-value, log-rank test) between the survival curves for the two patient groups stratified by the median of the rad-scores were calculated. In the training cohort, the rad score was a linear combination of each feature in the signature weighted by their corresponding CPHMs coefficients [12,22]. The coefficients and medians were locked and applied to the test cohort to calculate the rad-scores and stratify patients. Only the signature that yielded the lowest *p* value in the test cohort was retained. The C-index was then calculated using negative rad-scores, as rad-scores and survival time were negatively correlated.

## 3. Results

Supplementary Table S3 shows the mean and SD of the ICCs for conventional, b0, and b1 PH features for the RIDER dataset. The highest mean ICCs for conventional and PH features were 0.672 and 0.750, respectively (*p*-value = 1.15 × 10^−10^, Mann–Whitney U test). Owing to the higher mean ICCs, PH features were more repeatable than conventional ones.

Supplementary Figure S1 shows all the optimized α and the number of signature candidates for conventional and PH features in this study. The log-rank *p* values in the training and test cohorts of conventional, PH, and clinical signatures are detailed in Supplementary Figures S2–S4, respectively. Table 3 summarizes the *p*-values and C-indices for all signature types (Table 2) in this study.

**Figure 4:**
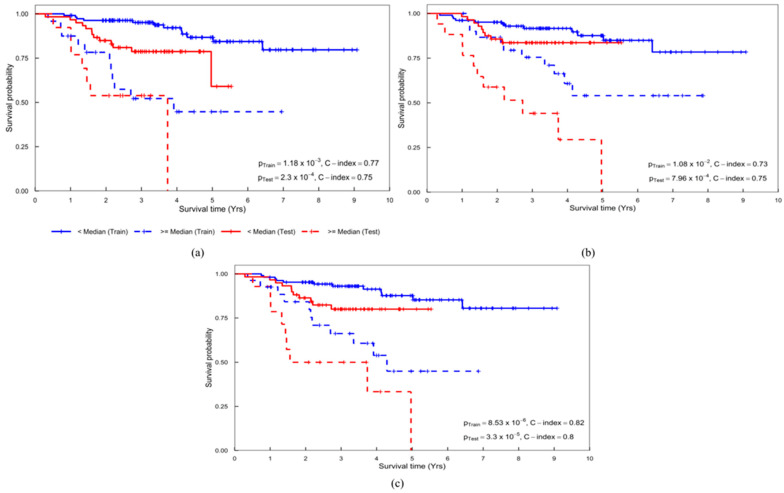
Kaplan–Meier curves obtained from the (**a**) clinical, (**b**) PH-PET, and (**c**) clinical + PH-PET signatures. PH-PET: persistent homology-positron emission tomography.

A combination of clinical and PH-PET signatures achieved the highest performance with a *p*-value of 3.30 × 10^−5^ and C-index (95% confidence interval [CI]) of 0.80 (0.78–0.82) in the test cohort (Table 3). Clinical signature solely yielded a *p*-value of 2.30 × 10^−4^ and C-index (95% CI) of 0.75 (0.73–0.78) in the test cohort (Table 3). PH-PET signature solely achieved a *p*-value of 7.96 × 10^−4^ and C-index (95% CI) of 0.75 (0.73–0.78) in the test cohort (Table 3). The above signatures are detailed in Supplementary Table S4, and Figure 4 illustrates the corresponding Kaplan–Meier curves.

Tumor volume achieved a *p*-value of 0.15 and C-index (95% CI) of 0.68 (0.67–0.70) in the training cohort, and *p*-value of 0.31 and C-index (95% CI) of 0.54 (0.50–0.57) in the test cohort. For HPV status, *p*-value and C-index in the training cohort were 0.12 and 0.59 (0.57–0.60), respectively, while those in the test cohort were 0.29 and 0.58 (0.56–0.59), respectively. The clinical + PH-PET signatures also outperformed tumor volume and HPV status.

## 4. Discussion

This study investigated the feasibility of using the PH features extracted from PET/CT images for prognostic prediction in patients with HN cancer. To the best of our knowledge, this is the first study to examine the potential of PH features on PET/CT images for prognostic prediction of patients with HN cancer.

The PH features proposed in this study could provide more intrinsic information on the underlying metabolic and morphological traits of tumors from the PET/CT images. The combination of the clinical and PH-PET signatures achieved the highest performance (Table 3). This result, thus, proved the feasibility of PH features and further agreed with Vallières et al. [13] that a combination of radiomic and clinical signatures could improve prognostic power. Furthermore, this study indicated that the contribution of metabolic and morphologic information obtained from PET/CT images in the prognostic prediction of patients with HN was in agreement with the work of El Naqa et al. [11].

We suggest the following clinical scenario in which a better treatment method can be selected using the system of the proposed approach. A physician can input PET/CT images of an HN cancer patient into the system to predict the high or low risk of death for the patient, before the patient receives treatment (radiotherapy or concurrent chemoradiotherapy). If the output of the system to a patient is low-risk, the physician may select radiotherapy or concurrent chemoradiotherapy. If the output is-high risk, the physician may not select that treatment.

Because of its ability to treat survival time as a continuous variable and account for patient censorship [23], the Coxnet algorithm enabled us to select signature candidates that were correlated with survival time. More importantly, this Coxnet algorithm also provided consistency between feature selection and model building using CPHMs; hence, any potential information loss could be avoided.

PH features (Table 3) also outperformed the independent predictors of tumor volume [33] and HPV status [35,36]. The small number of patients with available HPV status could be the main factor that deteriorates the performance of CPHMs. Hatt et al. [37] reported that texture analysis may provide more information when the tumor size is larger than 10 cm^3^. In our dataset, because the majority of tumors exceeded 10 cm^3^ (*n* = 193/207), PH features were found to have more prognostic power than tumor volume. This result is in agreement with the result of the studies by Vallières et al. [13] and Hatt et al. [37].

Our training and test cohorts included patients with heterogeneous cancer stages and treatment methods. PET/CT images were acquired at different institutions, vendors, tube voltages, exposures, slice thickness, and reconstruction filters [13]. However, PH signatures still outperformed conventional signatures (Table 3). Hence, PH features may be considered more robust than the conventional approaches when there are variations in cancer stages, treatment methods, and acquisition techniques.

Although only one dataset (*n* = 207) was used, a portion of the dataset (*n* = 73) was set aside as a completely independent test cohort. This division allowed us to perform an external validation of our predictive models, which could be considered a more accurate evaluation of model performance than an internal validation or random splitting of the dataset [38].

This study had several limitations. First, the relatively small number of patients used for training may have reduced the prognostic power of the predictive models. More patients should be included in future studies. Second, signatures constructed using a homogeneous dataset may differ from ours. Although an evaluation using an independent test cohort suggested that the PH technique could be robust against variations in cancer stages and treatment methods, more investigations regarding this effect on the final model need to be conducted. Third, variations in imaging protocols were not investigated. Since inter-scanner variability (time resolution, detector sensitivity correction, dead time correction, random and scatter coincidence corrections, attenuation correction, and image reconstruction) has been reported to affect conventional features extracted from CT [39,40] and PET images [41,42], the impact of these factors on PH features will be further examined in our future studies. Streak artifacts, which can decrease the performance of radiomic models [43], were not examined in this study. Despite favorable results, the effect of streak artifacts on PH features should be carefully examined in future studies. Finally, although a combination of radiomic signatures and HPV status has been reported to increase the prognostic value [12], we could not perform this task because HPV status was not fully available in our dataset (*n* = 83/207).

## 5. Conclusions

This study was the first trial to investigate the feasibility of a PH technique for FDG-PET/CT imaging to predict prognoses in patients with HN. PH features could capture topological information about metabolism and morphology of tumors that could be associated with the prognoses. Because heterogeneity is a common characteristic among tumors, we believe that this PH technique can be widely applied to other cancer types to improve prognostic prediction. Hence, personalized treatment could be further facilitated and patient survival lengthened.

## Figures and Tables

**Table 1:** Clinical variables of the patients in this study.

	Training Cohort (*n* = 134)	Test Cohort (*n* = 73)
Age [years] (median)	18–84 (62)	44–90 (64)
Sex (Male/Female)	103/31	54/19
Tumor volume [cm^3^] (median)	1.98–348.62 (37.06)	3.31–245.45 (36.42)
Human papilloma virus (HPV) status (positive/negative/no information)	41/24/69	16/2/55
T stage (T1/T2/T3/T4/T4A/T4B)	20/42/49/14/6/3	6/35/17/11/2/2
N stage (N0/N1/N2/N2A/N2B/N2C/N3/N3B)	31/19/31/7/27/15/4/0	7/11/29/0/9/10/6/1
M stage (M0)	134	73
TNM stage (I/II/IIB/III/IV/IVA/IVB)	2/11/1/36/0/76/8	0/4/1/10/36/17/5
Survival censorship (Event/Censor)	111/23	20/53
Site (Larynx/Oropharynx/ Nasopharynx/Hypopharynx)	22/94/16/2	6/55/6/6

**Table 2:** Thirteen types of signatures constructed in this study.

1 Clinical Signature	3 Conventional Signatures	3 PH Signatures	6 Combined Signatures
Clinical	Conventional CT	PH-CT	Conventional CT + clinical
	Conventional PET	PH-PET	Conventional PET + clinical
	Conventional PET/CT	PH-PET/CT	Conventional PET/CT + clinical
			PH-CT + clinical
			PH-PET + clinical
			PH-PET/CT + clinical

**Table 3:** Summary of *p*-values and C indices from the 13 types of signatures in this study.

	Training Cohort		Test Cohort	
	*p*-Value	C-Index (95% CI)	*p*-Value	C-Index (95% CI)
Clinical signature	1.18 × 10^−3^	0.77 (0.75–0.79)	2.30× 10^−4^	0.75 (0.73–0.78)
Conventional CT signature	2.03 × 10^−4^	0.75 (0.73–0.76)	2.24× 10^−3^	0.35 (0.32–0.39)
Conventional PET signature	9.65 × 10^−1^	0.53 (0.51–0.55)	2.32× 10^−2^	0.66 (0.63–0.69)
Conventional PET/CT signature	4.96 × 10^−3^	0.72 (0.70–0.74)	1.68× 10^−2^	0.71 (0.68–0.74)
PH-CT signature	1.62 × 10^−2^	0.64 (0.63–0.66)	1.39× 10^−3^	0.34 (0.32–0.36)
PH-PET signature	1.08 × 10^−2^	0.73 (0.71–0.75)	7.96× 10^−4^	0.75 (0.73–0.78)
PH-PET/CT signature	7.83 × 10^−4^	0.68 (0.66–0.69)	4.25× 10^−4^	0.66 (0.63–0.68)
Clinical + Conventional CT signature	4.46 × 10^−3^	0.8 (0.78–0.81)	3.52× 10^−2^	0.39 (0.36–0.43)
Clinical + Conventional PET signature	1.18 × 10^−3^	0.77 (0.75–0.79)	4.72× 10^−4^	0.75 (0.73–0.78)
Clinical + Conventional PET/CT signature	5.26 × 10^−4^	0.82 (0.81–0.83)	1.47× 10^−4^	0.79 (0.77–0.81)
Clinical + PH-CT signature	4.89 × 10^−3^	0.77 (0.75–0.79)	1.15× 10^−4^	0.73 (0.71–0.76)
Clinical + PH-PET signature	8.53 × 10^−6^	0.82 (0.81–0.83)	3.30× 10^−5^	0.80 (0.78–0.82)
Clinical + PH-PET/CT signature	3.79× 10^−4^	0.78 (0.76–0.79)	5.69× 10^−4^	0.78 (0.76–0.80)

## Data Availability

Publicly available datasets were analyzed in this study. This data can be found here: https://www.cancerimagingarchive.net/.

## Supplementary Materials

The following supporting information can be downloaded at: https://www.mdpi.com/article/10.3390/metabo12100972/s1, Figure S1: Optimized blending parameter  α of the Coxnet model (left) and number of selected signature candidates (right) for 6-, 7-, 8-, and 9- (a) Conventional (CT, PET, and PET/CT), (b) b0 and b1 PH-CT, (c) b0 and b1 PH-PET, and (d) b0 and b1 PH-PET/CT features; Figure S2: Log-rank p-values in the training and test cohorts of CPHMs built using conventional (a) CT, (b) PET, and (c) PET/CT signatures; Figure S3: Log-rank p-values in the training (left column) and test cohorts (right column) of CPHMs built using (a) PH-CT, (b) PH-PET, and (c) PH-PET/CT signatures; Figure S4: Log-rank p-values in the training and test cohorts of CPHMs built using clinical signatures; Table S1: Histogram-based and texture features used in this study [10,44,45,46,47,48,49,50,51,52]; Table S2: Conversion table for clinical variables; Table S3: Means and standard deviations of intra-class correlation coefficients for conventional, b1, and b1 features; Table S4: Best signatures from conventional and PH-based features.
