# Supplementary material for: Can Persistent Homology Features Capture More Intrinsic Information about Tumors from 18F-Fluorodeoxyglucose Positron Emission Tomography/Computed Tomography Images of Head and Neck Cancer Patients?
Source: Metabolites. 2022 Oct 14;12(10):972. doi: 10.3390/metabo12100972 (PMC9610853; doi:10.3390/metabo12100972)
Supplement: Supplementary file 1 [file metabolites-12-00972-s001.zip › metabolites-1955996-supplementary.pdf]

Article

Can persistent homology features capture more intrinsic structural information of tumors from CT and PET images of head-and-neck cancer patients?

Quoc Cuong Le [1], Hidetaka Arimura [2,*], Kenta Ninomiya[3], Takumi Kodama[3] and Tetsuhiro Moriyama [2]

[1] Ho Chi Minh City Oncology Hospital, Ho Chi Minh City, Vietnam
[2] Department of Health Sciences, Faculty of Medical Sciences, Kyushu University, Japan
[3] Department of Health Sciences, Graduate school of Medical Sciences, Kyushu University, Japan
[1] Institute of Mathematics for Industry, Kyushu University, Japan
* Correspondence: arimura.hidetaka.616@m.kyushu-u.ac.jp

Table S1. Histogram-based and texture features used in this study.

	Texture type	Reference(s)	Feature name
14 histogram-based features	-	-	Energy<br>Entropy<br>Kurtosis<br>Maximum<br>Mean<br>Mean absolute difference (MAD)<br>Median<br>Minimum<br>Range<br>Root mean square (RMS)<br>Skewness<br>Standard deviation (STD)<br>Uniformity<br>Variance
45 Texture features	Gray-level co-occurrence matrix (GLCM)	Haralick et al. [1]	Energy<br>Contrast<br>Entropy<br>Homogeneity<br>Correlation
		Haralick et al. [1], Assefa et al. [2]	Sum Average<br>Variance
		Thibault [3]	Dissimilarity
		Aerts et al. [4]	Auto correlation
	Gray-level run-length matrix (GLRLM)	Galloway [5]	Short run emphasis (SRE)<br>Long run emphasis (LRE)<br>Gray-level nonuniformity (GLN)<br>Run-length nonuniformity (RLN)<br>Run percentage (RP)
		Chu et al. [6]	Low gray-level run emphasis (LGRE)<br>High gray-level run emphasis (HGRE)
		Dasarathy and Holder [7]	Short run low gray-level emphasis (SRLGE)<br>Short run high gray-level emphasis (SRHGE)

			Long run low gray-level emphasis (LRLGE)<br>Long run high gray-level emphasis (LRHGE)
		Thibault et al. [8]	Gray-level variance (GLV)<br>Run-length variance (RLV)
	Gray-level size zone matrix (GLSZM)	Galloway [5], Thibault et al. [8]	Small zone emphasis (SZE)<br>Large zone emphasis (LZE)<br>Gray-level nonuniformity (GLN)<br>Zone-size nonuniformity (ZSN)<br>Zone percentage (ZP)
		Chu et al. [6], Thibault et al. [8]	Low gray-level zone emphasis (LGZE)<br>High gray-level zone emphasis (HGZE)
		Dasarathy and Holder [7], Thibault et al. [8]	Small zone low gray-level emphasis (SZLGE)<br>Small zone high gray-level emphasis (SZHGE)<br>Large zone low gray-level emphasis (LZLGE)<br>Large zone high gray-level emphasis (LZHGE)
		Thibault et al. [8]	Gray-level variance (GLV)<br>Zone-size variance (ZSV)
	Neighborhood gray-tone difference matrix (NGTDM)	Amadasun and King [9]	Coarseness<br>Contrast<br>Busyness<br>Complexity<br>Strength
	Neighboring gray-level dependence matrix (NGLDM)	Sun and Wee [10]	Small number emphasis (SNE)<br>Large number emphasis (LNE)<br>Number nonuniformity (NN)<br>Second moment (SM)<br>Entropy

Table S2. Conversion table for clinical variables.

T stage		N stage		TNM stage		HPV	
Character value	Numeric value	Character value	Numeric value	Character value	Numeric value	Character value	Numeric value
T0	1	N0	1	I	1	NA	Exclude
T1	2	N1	2	II	2	Negative	1
T2	3	N2	3	III	3	Positive	2
T3	4	N2a	4	IV	4		
T3a	5	N2b	5	IVA	5		
T4	6	N2c	6	IVB	6		
T4a	7	N3	7				
T4b	8	N3a	8				
		N3b	9				

Table S3. Means and standard deviations of intra-class correlation coefficients for conventional, b1, and b1 features.

Conventional features			b0 PH features			b1 PH features		
	Mean	SD		Mean	SD		Mean	SD
6 bit	0.672	0.229	6 bit_b0_0.0001	0.636	0.269	6 bit_b1_0.0001	0.688	0.218
7 bit	0.656	0.239	6 bit_b0_0.001	0.630	0.284	6 bit_b1_0.001	0.660	0.235
8 bit	0.651	0.238	6 bit_b0_0.01	0.636	0.275	6 bit_b1_0.01	0.654	0.241
9 bit	0.636	0.247	6 bit_b0_0.1	0.648	0.269	6 bit_b1_0.1	0.662	0.234
			6 bit_b0_1	0.750	0.198	6 bit_b1_1	0.749	0.199
			7 bit_b0_0.0001	0.665	0.260	7 bit_b1_0.0001	0.683	0.235
			7 bit_b0_0.001	0.678	0.254	7 bit_b1_0.001	0.671	0.241
			7 bit_b0_0.01	0.685	0.230	7 bit_b1_0.01	0.678	0.237
			7 bit_b0_0.1	0.693	0.252	7 bit_b1_0.1	0.696	0.232
			7 bit_b0_1	0.747	0.215	7 bit_b1_1	0.693	0.221
			8 bit_b0_0.0001	0.690	0.252	8 bit_b1_0.0001	0.653	0.256
			8 bit_b0_0.001	0.682	0.236	8 bit_b1_0.001	0.648	0.255
			8 bit_b0_0.01	0.664	0.263	8 bit_b1_0.01	0.611	0.268
			8 bit_b0_0.1	0.729	0.218	8 bit_b1_0.1	0.598	0.283
			8 bit_b0_1	0.718	0.227	8 bit_b1_1	0.684	0.239
			9 bit_b0_0.0001	0.680	0.258	9 bit_b1_0.0001	0.661	0.227
			9 bit_b0_0.001	0.726	0.224	9 bit_b1_0.001	0.658	0.237
			9 bit_b0_0.01	0.731	0.222	9 bit_b1_0.01	0.689	0.225
			9 bit_b0_0.1	0.722	0.228	9 bit_b1_0.1	0.687	0.221
			9 bit_b0_1	0.706	0.243	9 bit_b1_1	0.686	0.223

Table S4. Best signatures from conventional and PH-based features.

Conventional CT	Conventional PET	Conventional PET/CT
CT_ LHH_NGTDM_Busyness	PET_LHH_NGTDM_Strength	CT_LHL_GLSZM_GLV
CT_Hist_Mean		CT_Hist_STD
CT_GLSZM_LZLGE		CT_LHH_GLSZM_LZLGE
CT_LHH_GLRLM_SRLGE		
CT_HHL_GLSZM_GLV		
CT_HLH_Hist_Kurtosis		
**PH-CT**	**PH-PET**	**PH-PET/CT**
CT_ b1_HLH_GLCM_Correlation	PET_ b0_Hist_Min	PET_b0_HHL_Hist_Mean
	PET_ b0_LLL_GLSZM_SZHGE	CT_b1_HLH_Hist_Mean
	PET_ b0_HHL_GLSZM_LGZE	PET_b1_LHL_GLCM_AutoCorrelation
	PET_ b0_HHL_GLSZM_LZE	

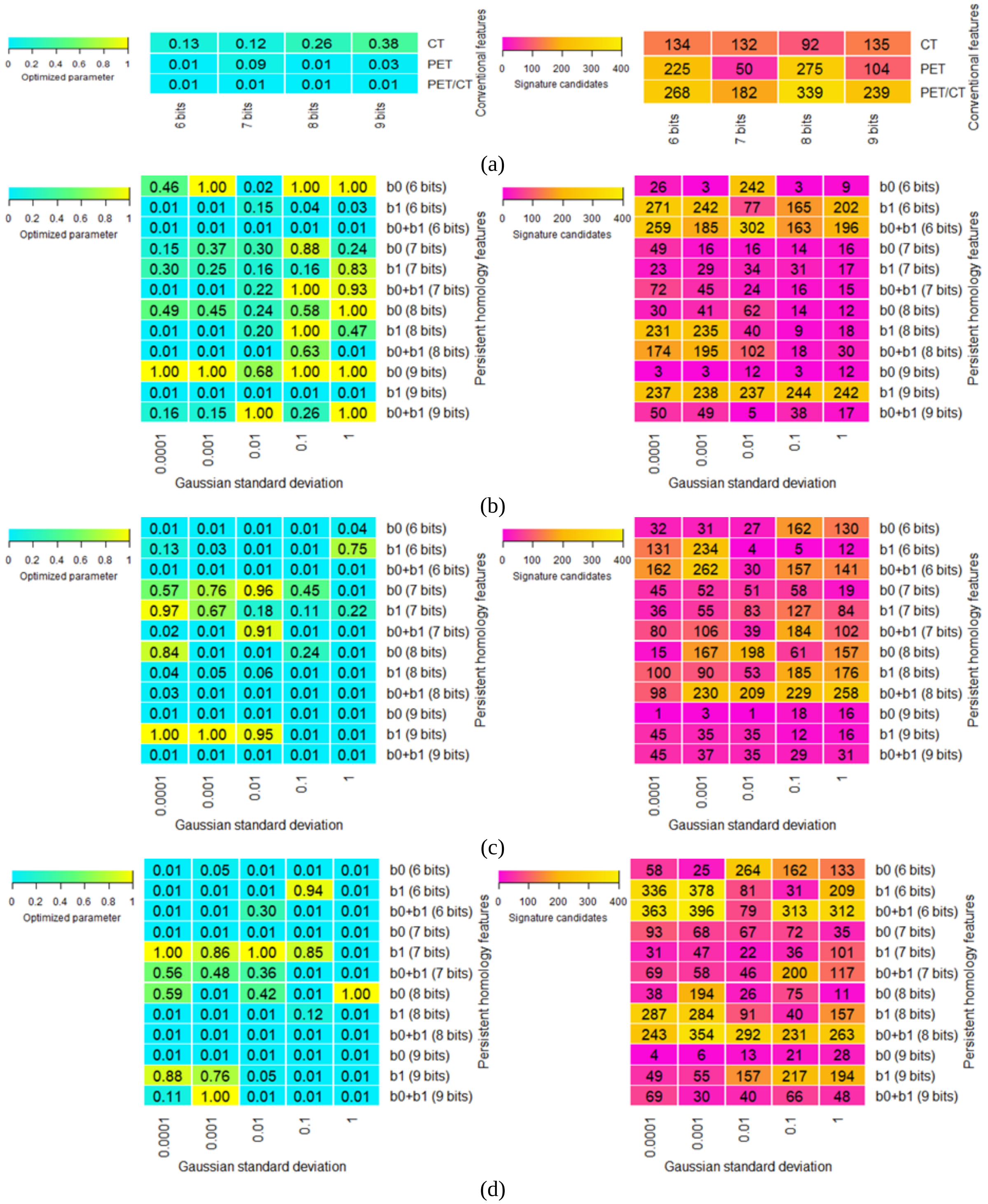

Figure S1. Optimized blending parameter α of the Coxnet model (left) and number of selected signature candidates (right) for 6-, 7-, 8-, and 9- (a) Conventional (CT, PET, and PET/CT), (b) b0 and b1 PH-CT, (c) b0 and b1 PH-PET, and (d) b0 and b1 PH-PET/CT features.

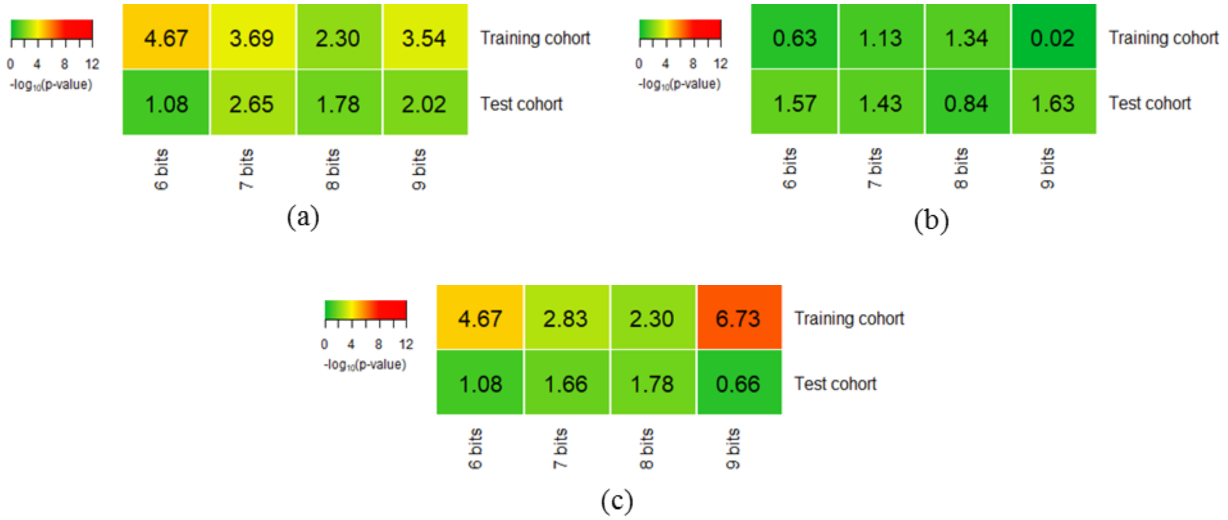

Figure S2. Log-rank p-values in the training and test cohorts of CPHMs built using conventional (a) CT, (b) PET, and (c) PET/CT signatures.

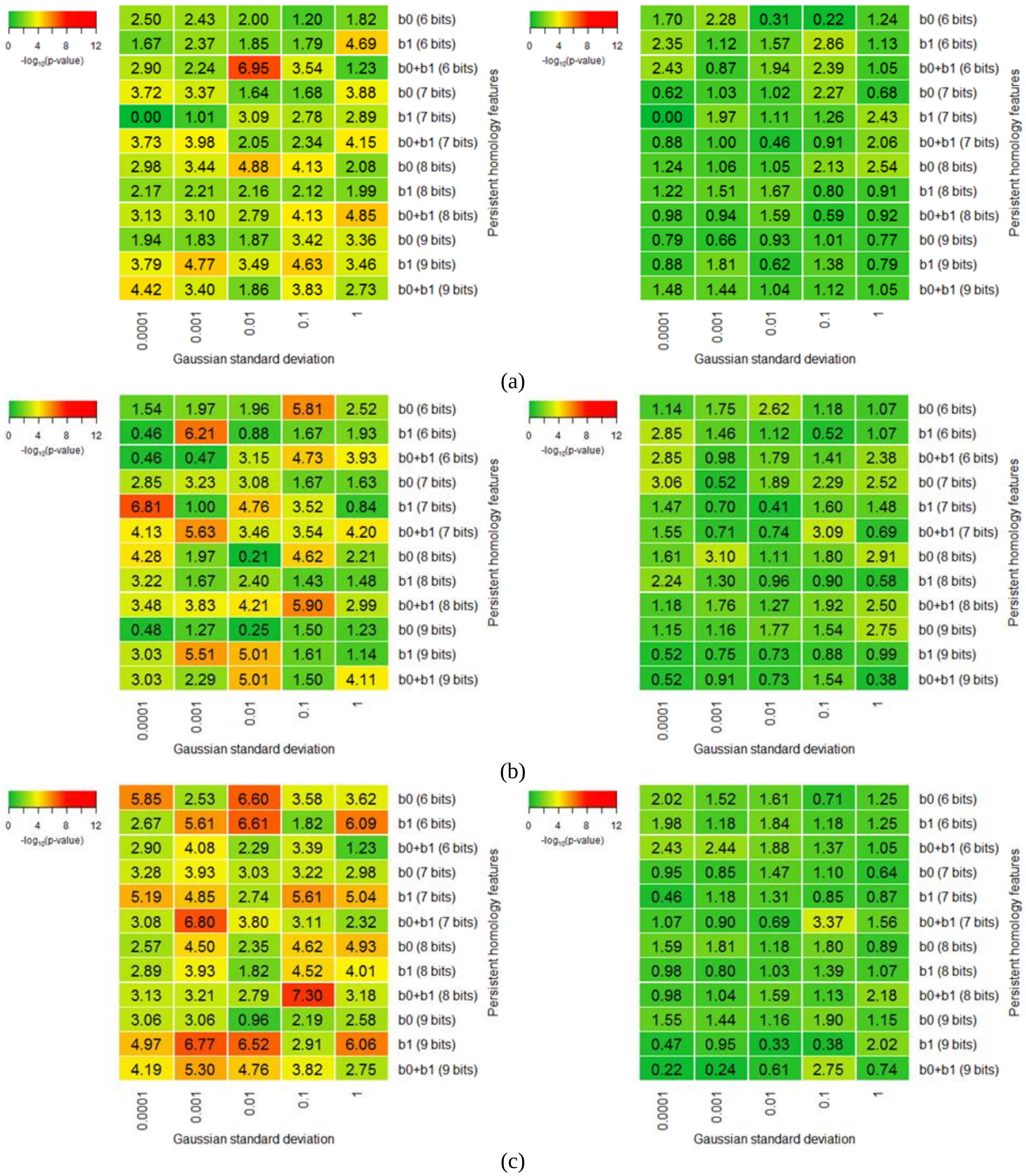

Figure S3. Log-rank p-values in the training (left column) and test cohorts (right column) of CPHMs built using (a) PH-CT, (b) PH-PET, and (c) PH-PET/CT signatures.

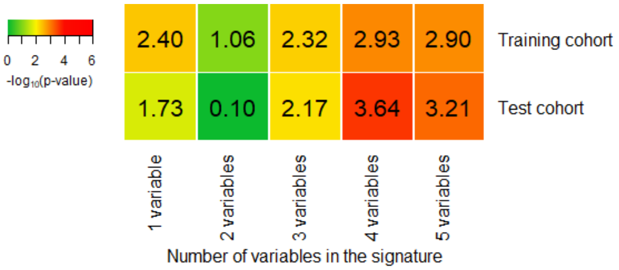

Figure S4. Log-rank p-values in the training and test cohorts of CPHMs built using clinical signatures.

REFERENCES

1. Haralick, R.M.; Shanmugam, K.; Dinstein, I. Textural features for image classification. *IEEE Trans. Syst. Man Cybern.* **1973** 3:610-621. 10.1109/TSMC.1973.4309314

2. Assefa, D.; Keller, H.; Ménard, C.; et al. Robust texture features for response monitoring of glioblastoma multiforme on T1-weighted and T2-FLAIR MR images: A preliminary investigation in terms of identification and segmentation. *Med. Phys.* **2010** 34(4): 1722-36. https://doi.org/10.1118/1.3357289

3. Thibault, G. Indices de formes et de textures: de la 2D vers la 3D. Application au classement de noyaux de cellules *PhD Thesis* University AIX-Marseille, France, 2009.

4. Aerts, H.J.W.L.; Velazquez, E.R.; Leijenaar, R.T.H.; et al. Decoding tumour phenotype by noninvasive imaging using a quantitative radiomics approach. *Nat. Commun.* **2014** 2014; 5: 4006. https://doi.org/10.1038/ncomms5006

5. Galloway, M.M. Texture analysis using gray level run lengths. *Comput. Graph. Image Process.* **1975** 4:172-179. https://doi.org/10.1016/s0146-664x(75)80008-6

6. Chu, A.; Sehgal, C.; Greenleaf, J. Use of gray level distribution of run lengths for texture analysis. *Pattern Recognit. Lett.* **1990** 11:415-419. https://doi.org/10.1016/0167-8655(90)90112-f

7. Dasarathy, B.; Holder, E. Image characterizations based on joint gray-level run length distributions. *Pattern Recogn. Lett.* **1991** 12:497-502. https://doi.org/10.1016/0167-8655(91)80014-2

8. Thibault, G.; Fertil, B.; Navarro, C.; et al. Texture indexes and gray level size zone matrix: application to cell nuclei classification Pattern Recognition and Information Processing (PRIP), Minsk, Belarus, 2009.

9. Amadasun, M.; King, R. Textural features corresponding to textural properties. *IEEE Trans. Syst. Man Cybern.* **1989** 19:1264–1274. https://doi.org/10.1109/21.44046

10. Sun, C.; Wee, W.G. Neighboring gray level dependence matrix for texture classification. *Comput. Vision Graph Image Process* **1983** 23:341-352. https://doi.org/10.1016/0146-664x(82)90093-4
